# Gray matter alteration in medication overuse headache: a coordinates-based activation likelihood estimation meta-analysis

**DOI:** 10.1007/s11682-022-00634-9

**Published:** 2022-02-10

**Authors:** Wenjia Chen, Hui Li, Xiaoyan Hou, Xize Jia

**Affiliations:** 1 Headache Sub-department of Neurology Department, Second Affiliated Hospital of Guangzhou University of Chinese Medicine/Guangdong Provincial Hospital of Chines Medicine, Guangzhou, People’s Republic of China; 2 Research Group of Standardization of Chinese Medicine, Second Affiliated Hospital of Guangzhou University of Chinese Medicine/Guangdong Provincial Hospital of Chines Medicine, Guangzhou, People’s Republic of China; 3 Radiology Department, Second Affiliated Hospital of Guangzhou University of Chinese Medicine/Guangdong Provincial Hospital of Chinese Medicine, Guangzhou, People’s Republic of China; 4 Department of Psychology Science, Hangzhou Normal University, Hangzhou, People’s Republic of China

**Keywords:** Medication overuse headache, ALE, Gray matter, VBM, Detoxification

## Abstract

Medication overuse headache (MOH) is a prevalent secondary headache, bringing heavy economic burden and neuropsychological damage. Neuroimaging studies on the disease reported divergent results. To merge the reported neuroimaging alterations in MOH patients and explore a pathophysiological mechanism of this disorder. A meta-analytic activation likelihood estimation (ALE) analysis method was used. We systematically searched English and Chinese databases for both morphological and functional neuroimaging studies published before Nov 18, 2021. Reported altered brain regions and the stereotactic coordinates of their peaks were extracted and pooled by GingerALE using Gaussian probability distribution into brain maps, illustrating converged regions of alteration among studies. We identified 927 articles, of which five studies on gray matter changes, using voxel-based morphometry (VBM) were eventually included for ALE analysis, with 344 subjects and 54 coordinates put into GingerALE. No functional magnetic resonance imaging (fMRI) or positron emission topography (PET) studies were included for pooling. Compared with healthy controls (HCs), MOH featured increased gray matter density in midbrain, striatum, cingulate, inferior parietal cortex and cerebellum (*P* < 0.001 uncorrected), whereas decreased gray matter density in orbitofrontal cortex (*P* < 0.05, family-wise error), frontal, insular and parietal cortices (*P* < 0.001 uncorrected). Withdrawal of analgesics led to decreased gray matter density in superior temporal gyrus, cuneus, midbrain and cerebellum (*P* < 0.001 uncorrected). This meta-analysis confirmed that medication overuse headache is associated with morphologic alteration in the reward system, the prefrontal cortex and a reversible modification in the pain network. Further functional imaging paradigms and longitudinal studies are required for a more definite conclusion and a causal mechanism.

## Introduction

Medication overuse headache (MOH) is a chronic headache that occurs on 15 or more days per month for over three months with overuse of acute or symptomatic headache medications for more than 10 or 15 days per month depending on overdosed medication (Headache Classification Committee of the International Headache Society, 2018). It was reported MOH patients worldwide exceeded 800 million in 2017 (James et al., 2018). As much as 4% of the worldwide population is overusing painkillers, of which about 1% suffer from overdose headache (Diener & Limmroth, 2004). About 53% of chronic migraine (CM) patients repeatedly overuse analgesics (Westergaard et al., 2014). MOH can be caused by overdosing ergots, triptan, barbiturates, opioids, nonsteroidal anti-inflammatory drugs (NSAIDs, e.g. aspirin, acetaminophen, etc.), or a combination of these drugs. The harm of MOH is not restricted to persistent pain and heavy economic burden, but also the toxicity of the drug itself. Excessive ergotamine can cause sensory nerve damage, central cognitive function damage, and decreased ductility of the craniocerebral artery wall, causing psychological dysfunction (such as severe pain) (Cevoli et al., 2017). Long time exposure to triptans in animals enhanced calcitonin gene-related peptide and nitric oxide system activity, resulting in persistent allodynia (De Felice et al., 2009).

The pathogenesis of MOH, which remains elusive, involves central sensitization(Ayzenberg et al., 2006), dysfunction of the endogenous serotonin system dysfunction(Reuter et al., 2004), etc. In the past decade, multiple scholars explored with magnetic resonance imaging (MRI) the neuroimaging changes of MOH but obtained divergent results. Lai et al. (Lai et al., 2016) reported that MOH patients, compared with healthy controls (HCs), displayed gray matter atrophy in the rectal gyrus, inferior frontal gyrus, middle frontal gyrus, and precuneus. While Mehnert et al. (Mehnert et al., 2018) discovered that MOH related gray matter atrophy located in the medial orbital gyrus, hippocampus, inferior frontal gyrus, and precuneus. However, several researchers did not find gray matter changes in MOH relative to HCs (Beckmann et al., 2018; Chanraud et al., 2014). Disparity among studies is apparent.

The purpose of this study was to explore a pattern of common neuroimaging change in MOH patients, with the help of a novel meta-analytic activation likelihood estimation (ALE) algorithm (Eickhoff et al., 2009, 2012; Turkeltaub et al., 2012), by merging the reported neuroimaging abnormalities from different studies, shedding light on a neurophysiologic mechanism of this disorder.

## Methods

### Literature search and selection

In this study, we adopted the meta-analysis definition embraced by the Cochrane Collaboration and followed the *Preferred Reporting Items for Systematic Reviews and Meta-Analyses* (PRISMA) *Statement* guidelines. A systematic search was carried out to thoroughly include all relevant studies on MOH published before Nov. 18, 2021, using all available imaging techniques, including MRI, blood-oxygen-level dependent (BOLD)-functional MRI (fMRI) or positron emission tomography (PET), that are compatible for ALE. The query terms were as follows:

① analgesic overuse OR medication overuse OR medication overuse headache(MeSH term)
② headache or migraine(in title or abstract)
③ positron emission tomography or PET(in title or abstract)
④ MRI OR voxel-based OR structural OR cortical OR morphometric OR morphometry(in title or abstract)
⑤ fMRI OR functional connectivity OR functional connection OR regional homogeneity OR BOLD(in title or abstract)
⑥ ①AND②AND(③OR④OR⑤)

Three English databases (PubMed, Web of Science, EmBase) and three Chinese databases (China National Knowledge Infrastructure, Wanfang and China Biology Medicine disc) were queried. Inclusion criteria for eligible literature are as follows: (1) original clinical studies on human; (2) participants included patients diagnosed with MOH according to International Classification of Headache Disorders (ICHD); (3a) voxel-, volume-based gray matter analyses with T1 weighted imaging (T1WI) data; (3b) BOLD MRI studies; (3c) PET studies; (4) parameters of neuroimaging acquisition was reported; (5) results were reported using Montreal Neurological Institute (MNI) or Talairach coordinates; (6) studies published in Chinese or English in peer-recognized academic journals. The exclusion criteria of literature were: (1) duplicate reports of an included study; (2) conference reports; (3) studies without a comparing group; (4) single studies that cannot be pooled due to a lack of another matched study using comparable design; (5) studies yielding negative findings in which no coordinate was available.

Literature selection was performed by two neurology researchers, during which disagreement was resolved by the third researcher. Afterwards data from the included studies were extracted into spreadsheets and proofread by a group of researchers. Relevant information that was extracted included: (1) publication information (title, published journal, year of publication), (2) demographic information (number of subjects in each group, gender ratio, average age, handedness), (3) clinical information (diagnostic criteria, course of headache, time of medication overuse, types of overused medication), (4) technical information of neuroimaging (scanning techniques, scanned regions, magnetic field intensity, slice thickness), (5) analyzing methods of imaging data (spatial coordinates of vertices, types of coordinate system, methodology, software used and its version, methods for multiple comparison correction, and the diameter of Gaussian kernel).

### Statistical analysis

We adopted in our statistical analysis the ALE algorithm, a novel quantitative voxel-based method that can be used to estimate consistent change of gray matter (or functional image) from an array of imaging studies which reported peaks of gray matter alteration or functional activation of statistical significance (Laird et al., 2005). ALE requires that peak foci of clusters be reported in stereotactic coordinates (in “x,y,z” format). All reported foci were retrieved from articles and imported into the software. ALE approach, the technique of which has been described (Laird et al., 2005; Turkeltaub et al., 2002), models each alteration focus as the center of a spherical Gaussian probability distribution. In the updated version of ALE algorithm (GingerALE 3.0.2 http://brainmap.org) (Eickhoff et al., 2009), all reported foci (coordinates of maximum activation) for a given study are modelled as the peaks of stereotactic Gaussian probability distribution. A “modelled activation” (MA) map is computed, representing a summary of the coordinates from the specific study. ALE values are then calculated on a voxel-by-voxel basis by taking the union of these individual MA maps, with higher ALE value indicating more significance in a voxel. This revised analysis tests for convergence between studies (which is random-effects) rather than foci (which is fixed-effects).

Statistical significance of our analysis was assessed with a *P*-threshold corrected for comparisons using the family-wise error (FWE) (Eickhoff et al., 2012; Genovese et al., 2002) which is, and an uncorrected *P* which is more liberal. The results are presented at *P* < 0.05 with 1000 permutations FWE corrected for multiple comparisons, and an uncorrected *P* < 0.001 set with a minimum cluster volume of 100 mm^3^. Each thresholded ALE map produced in MNI space was overlaid using MRIcron and MRIcroGL software onto the International Consortium for Brain Mapping(ICBM)-152 template.

## Results

**Fig. 1:**
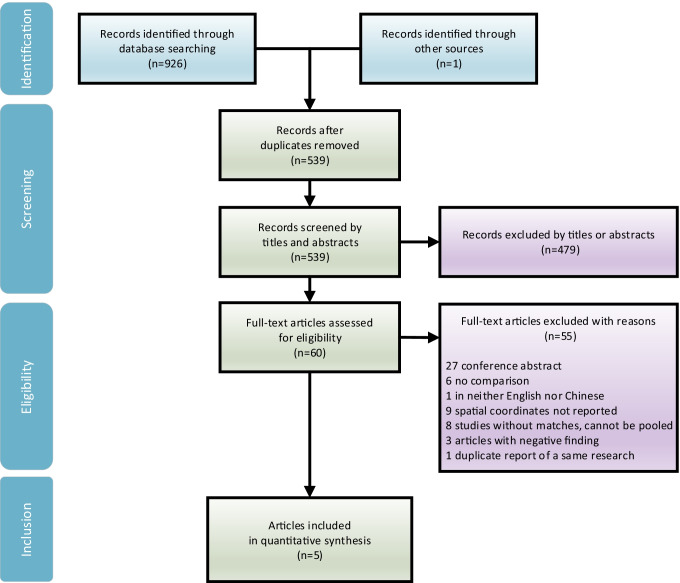
Literature selection following *Preferred Reporting Items for Systematic Reviews and Meta-Analyses* flowchart

Altogether we identified 927 articles, among which one article was found from reference of reviews, yielding five VBM studies into ALE quantitative synthesis after the inclusion and exclusion criteria were applied (see Fig. 1, Tables 1 and 2). All six identified fMRI studies were unfortunately excluded for they used disparate and incomparable methods of neuroimages statistical processing. The only two PET studies on MOH did not enter quantitative ALE synthesis for they set different control groups. The flowchart of literature selection following PRISMA statement is shown in Fig. 1. We put into GingerALE a total of 54 coordinates of vertices reported by the five included studies, which constructed two ALE analyses.

**Table 1:** Demographic and clinical information of included studies

Included studies	Number of subjects	Age (years) $\overline{{\varvec{x}}}\pm {\varvec{S}}{\varvec{D} }$	Male/female	Handedness	Duration of headache (years)	Overused medication (number of subjects)	Frequency of analgesics intake (days/month)
Riederer et al., 2012	MOH 29 Paired HC 29	41.4 ± 12.7 41.7 ± 12.8	7/22	Righthanded	15.1 ± 11.0 NA	Simple analgesics(21), Combination analgesics(3), Triptans(20), Opioids(3)	22.7 ± 7.7
Chen et al., 2018	MOH 36 HC 32	42.47 ± 9.34 41.34 ± 10.89	5/31 12/20	Righthanded	17.81 ± 5.81 NA	Not reported	NA
Lai et al., 2016	MOH 33 Paired HC 33	40.2 ± 10.0 39.7 ± 11.1	6/27	Righthanded	18.4 ± 10.4	NSAIDs(26), Triptans(1), Ergotamine(2), Opioids(2), Unknown(7)	24.7 ± 6.65 -
Mehnert et al., 2018	MOH 18 Paired HC 18	36 ± 14 34 ± 9	5/13	Righthanded	20 ± 14 -	NSAID(12), Triptans(5), Both(1)	21 ± 4 -
Riederer et al., 2013	Effective withdrawal 11 Ineffective withdrawal 11 (paired)	41.8 ± 9.1 44.3 ± 12.0	2/9	Righthanded	20.1 ± 12.2 19.0 ± 10.0	Simple analgesics(7), Triptans(6), Combinations(9)	6.4 ± 4.9 12.8 ± 9.4

*MOH* medication overuse headache; *HC* healthy control; *NSAID* non-steroidal inflammatory drug; *NA* not available

**Table 2:** Technical information of scanning and analyzing methods of imaging data used by included studies

Included studies	Scanning /magnetic field strength	Statistical Analysis	Software	Correction for multiple comparison
Riederer et al., 2012	MR T1WI/3 T	VBM	SPM8	FDR *P* < 0.05
Chen et al., 2018	MR T1WI/3 T	VBM	SPM (version unspecified)	FDR *P* < 0.05
Lai et al., 2016	MR T1WI/1.5 T	VBM	SPM8 (GLM Flex toolbox)	FWE *P* < 0.05
Mehnert et al., 2018	MR T1WI/3 T	VBM	CAT12	FWE *P* < 0.05
Riederer et al., 2013	MR T1WI/3 T	VBM	VBM8, SPM8	FWE *P* < 0.05

*MOH* Medication overuse headache; *HC* health controls; *SPM* Statistical Parametric Mapping; *FDR* false discovery rate; *FWE* family-wise error; *MR* magnetic resonance; *T1WI* T1 weighted image; *VBM* voxel-based morphometry; *FWE* family-wise error

**Fig. 2:**
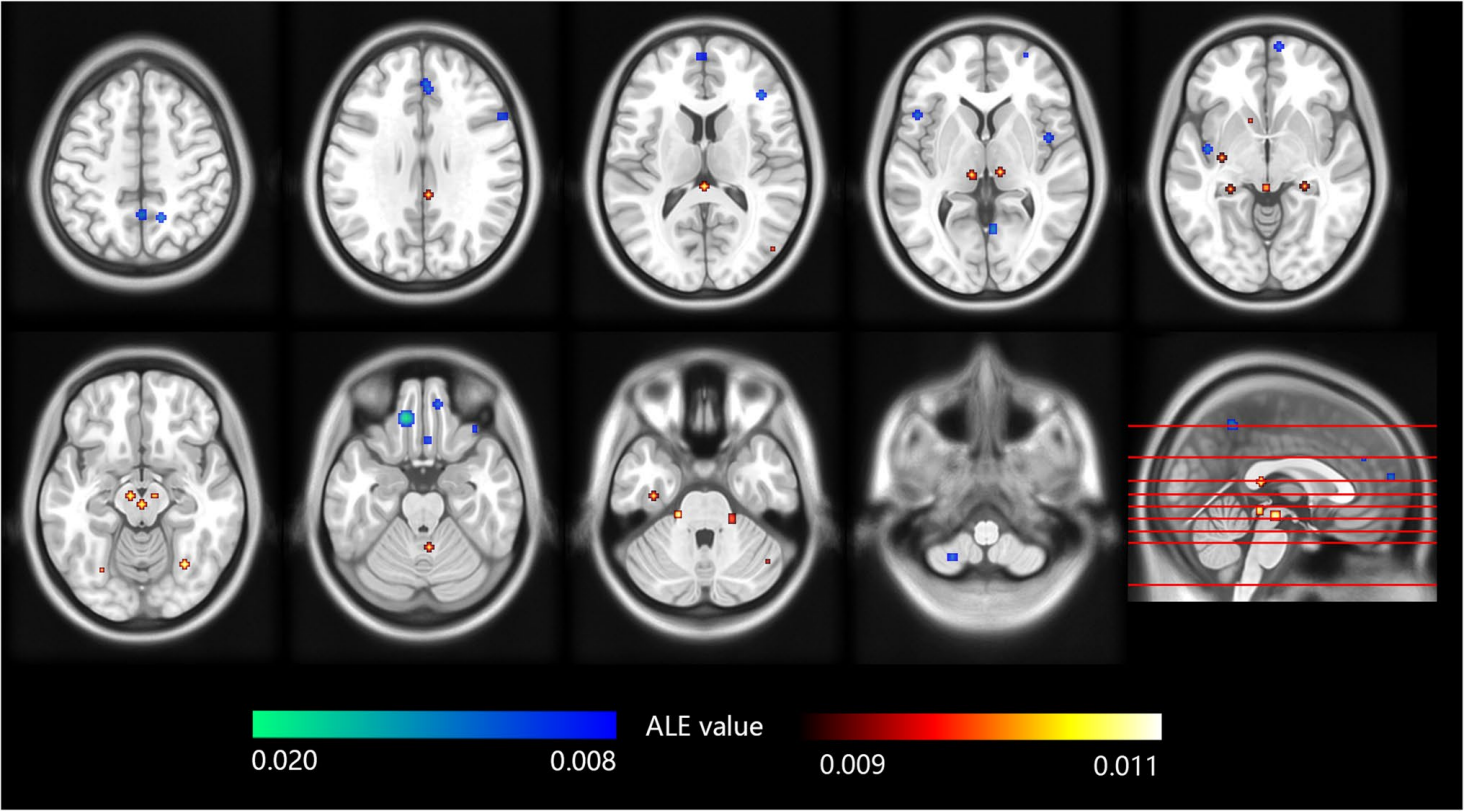
Brain regions that reported increased or decreased gray matter density in MOH patients compared with healthy controls by GingerALE. The color bars indicate ALE value of clusters. Red-yellow indicates increased gray matter density, while blue-indigo indicates decreased gray matter density. The left of the graph corresponds to the left side of patients

**Fig. 3:**
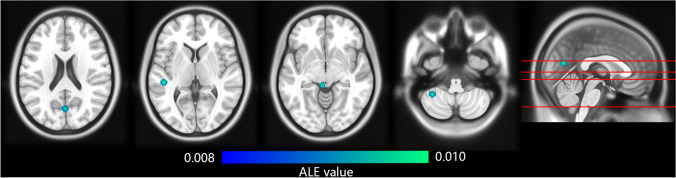
Brain regions that reported decreased gray matter density in detoxified MOH patients compared with baseline by GingerALE. The color bar indicates ALE value of clusters. The left of the graph corresponds to the left side of patients

Firstly, four studies on gray matter change of MOH compared with HCs were included in ALE, with 286 subjects and 50 vertices put in. It was reported that increased gray matter density in MOH patients located in midbrain, striatum, cingulate, inferior parietal cortex and cerebellum (*P* < 0.001 uncorrected). On the other hand, decreased gray matter density were reported in orbitofrontal cortex (*P* < 0.05, FWE corrected), frontal, insular and parietal cortices (*P* < 0.001 uncorrected; see Tables 3 and 4, Figs. 2 and 3).

**Table 3:** Brain regions that reported increased gray matter density in MOH patients compared with healthy controls by GingerALE

Increased gray matter density						Contributing studies
Brain areas	BA^a^	MNI coordinates			ALE (× 10^–3^)	
		X	Y	Z		
Midbrain						
PAG	-	0	-32	12	10.05	Riederer et al., 2012
L ventral lateral PAG	-	-2	-32	-8	9.48	Chen et al., 2018
L Ventral tegmental area	-	0	-22	-12	10.20	Chen et al., 2018
L substantia nigra	-	-8	-16	-12	10.20	Chen et al., 2018
L thalamus	-	-10	-24	2	10.05	Riederer et al., 2012
Trigeminal root entry zone						
L trigeminal root entry zone	-	-20	-28	-30	9.13	Chen et al., 2018
R trigeminal root entry zone	-	19	-32	-29	9.47	Chen et al., 2018
Striatum						
R ventral striatum/thalamus	-	10	-22	4	10.05	Riederer et al., 2012
R ventral striatum /substantia nigra	-	10	-16	-12	9.83	Chen et al., 2018
L ventral striatum	-	-12	14	-8	10.05	Riederer et al., 2012
R ventral striatum	-	10	18	-10	10.05	Riederer et al., 2012
L putamen	-	-32	-12	-4	10.05	Riederer et al., 2012
Cingulate g						
R posterior cingulate g	31	4	-38	30	10.05	Riederer et al., 2012
L middle cingulate g	24	-4	-6	36	10.05	Riederer et al., 2012
R inferior parietal cortex	-	48	-76	10	10.05	Riederer et al., 2012
Temporal lobe						
L fusiform g	37	-28	-68	-12	10.05	Riederer et al., 2012
	37	-36	-16	-30	10.05	Riederer et al., 2012
R fusiform g	37	30	-64	-14	10.05	Riederer et al., 2012
L hippocampus	-	-26	-34	-4	10.05	Riederer et al., 2012
R hippocampus	-	26	-32	-4	10.05	Riederer et al., 2012
Cerebellum						
R inferior cerebellum/cerebellar tonsil	-	34	-66	-46	10.05	Riederer et al., 2012
R inferior cerebellum	-	44	-62	-34	10.05	Riederer et al., 2012
R inferior cerebellum/ cerebellar tonsil	-	12	-56	-44	10.05	Riederer et al., 2012
R cerebellar vermis	-	4	-52	-24	10.05	Riederer et al., 2012
L inferior cerebellum/ cerebellar tonsil	-	-16	-52	-46	10.05	Riederer et al., 2012

*MNI* Montreal Neurological Institute; *L* left; *R* right; *g* gyrus; *ALE* Activation Likelihood Estimation; *PAG* periaqueductal gray; *BA* Brodmann Area

^a^ Some of the brain regions are not within a specific Brodmann Area

**Table 4:** Brain regions that reported decreased gray matter density in MOH patients compared with healthy controls by GingerALE

Regions with decreased gray matter density						Contributing studies
Brain areas	BA^b^	MNI Coordinates			ALE (× 10^–3^)	
		X	Y	Z		
Frontal lobe						
R medial orbital g	11	10	48	-22	9.47	Mehnert et al., 2018
L medial orbital g^a^	11	-12	38	-24	18.83	Mehnert et al., 2018; Lai et al., 2016
R orbital g	10	8	66	-4	10.05	Riederer et al., 2012
R rectal g	11	2	24	-26	9.45	Lai et al., 2016
L superior frontal g	46	-2	60	14	9.45	Lai et al., 2016
	46	28	60	0	10.05	Riederer et al., 2012
R medial frontal g	9	3	38	28	8.68	Riederer et al., 2012
	9	42	46	18	9.11	Lai et al., 2016
L inferior frontal g	47	-48	18	2	10.05	Lai et al., 2016
	47	-48	32	-10	9.80	Lai et al., 2016
R inferior frontal g	47	36	31	-20	9.80	Lai et al., 2016
	47	40	32	12	9.47	Mehnert et al., 2018
R frontal operculum	9	56	17	27	9.45	Lai et al., 2016
L precentral g	6	-30	-9	66	9.80	Lai et al., 2016
L insula	13	-42	-6	-6	10.05	Riederer et al., 2012
R insula	13	44	2	4	10.05	Riederer et al., 2012
L precuneus	7	0	-52	52	10.06	Riederer et al., 2012
R precuneus	7	10	-62	46	9.34	Lai et al., 2016
	7	14	-54	50	9.23	Mehnert et al., 2018
L superior occipital g/cuneus	19	-15	-98	20	9.80	Lai et al., 2016
R lingual g	30	5	-62	3	9.45	Lai et al., 2016
R hippocampus	-	30	-21	-9	8.82	Mehnert et al., 2018
L cerebellum	-	-24	-59	-62	9.80	Lai et al., 2016

*MNI* Montreal Neurological Institute; *L* left; *R* right; *g* gyrus; *ALE* Activation Likelihood Estimation; *PAG* periaqueductal gray; *BA* Brodmann Area

^a^ Family-wise error corrected, *P* < 0.05

^b^ Some of the brain regions are not within a specific Brodmann Area

In another ALE analysis, two studies comparing pre- and post-withdrawal of overused medication were also pooled using ALE. Four coordinates from 58 subjects were put into ALE, which reported that, after detoxification, gray matter density decreased in superior temporal gyrus, cuneus, midbrain and cerebellum (*P* < 0.001 uncorrected; see Table 5, Fig. 3).

**Table 5:** Reported brain regions with decreased gray matter density in detoxified MOH patients compared with baseline by GingerALE

Brain areas	BA^a^	MNI coordinates			ALE (× 10^–3^)	Contributing studies
		X	Y	Z		
L superior temporal g	22	-48	-30	3	9.47	Mehnert et al., 2018
L cuneus	17	0	-70	18	9.14	Mehnert et al., 2018
L midbrain	-	-5	-34	-8	8.33	Riederer et al., 2013

*MNI* Montreal Neurological Institute; *L* left; *g* gyrus; *ALE* Activation Likelihood Estimation; *BA* Brodmann Area

^a^ Some of the brain regions are not within a specific Brodmann Area

## Discussion

**Fig. 4:**
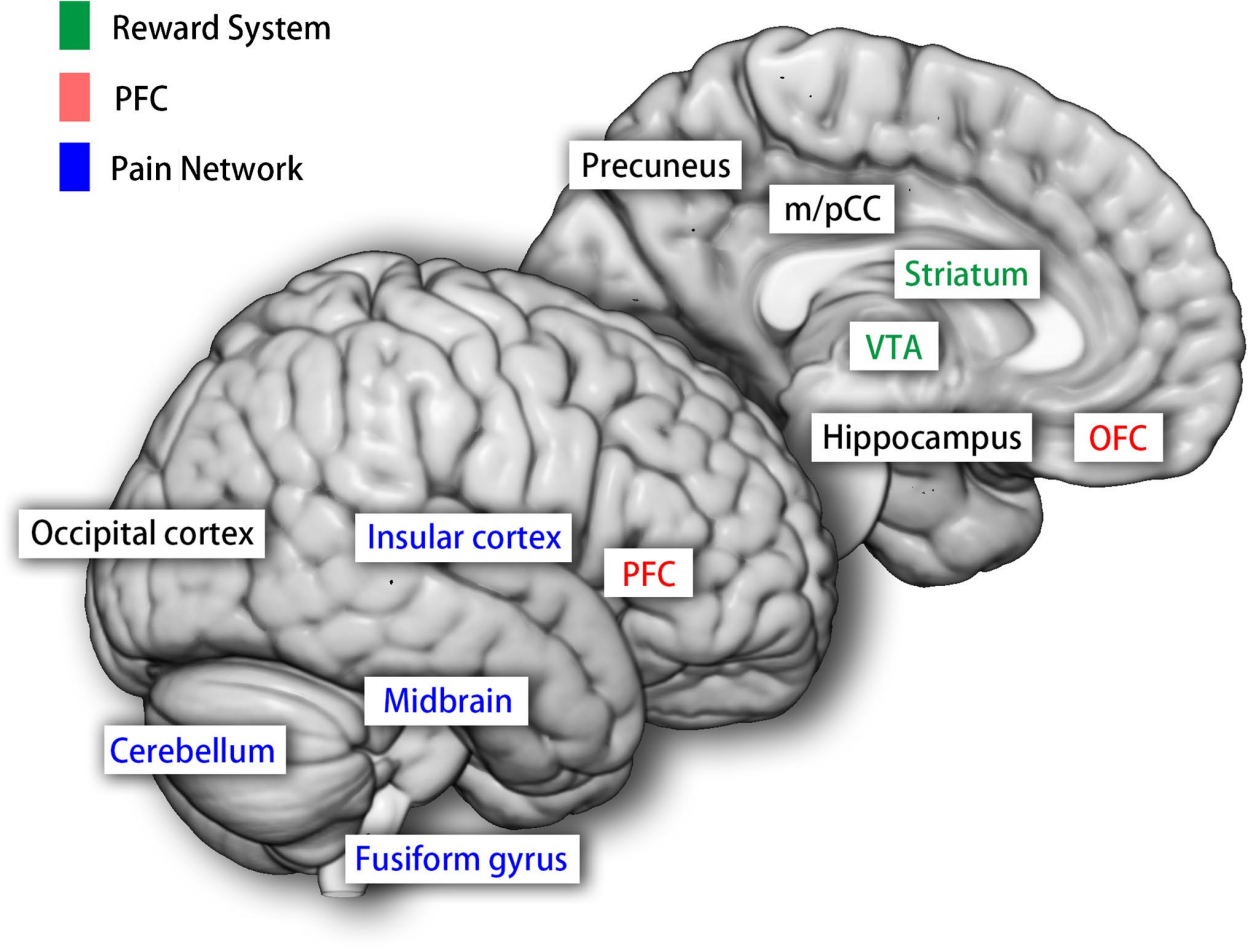
Illustration of altered networks discovered by meta-analytical activation likelihood estimation on gray matter alteration in medication overuse headache patients. PFC, prefrontal cortex; OFC, orbitofrontal cortex; VTA, ventral tegmental area; m/pCC, middle/posterior cingulate cortex

This research is the first meta-analytic ALE study on MOH so far. Based on our ALE study on the morphological alteration of MOH patients, it is confirmed that MOH is a secondary headache disorder closely associated with changes among multiple brain regions and networks. We observed abnormal gray matter in multiple regions including subcortical nuclei, brain stem and cerebellum. Gray matter of midbrain, cerebellum, cuneus and superior temporal gyrus decreased after detoxification, indicating some of the MOH-related gray matter increase may be reversible. The ALE method helped us in combining stereotactic coordinates of peaks of gray matter change from MOH neuroimaging studies, thus revealed that abnormal brain areas related to MOH are mainly distributed in the pain network, reward system, and the prefrontal area, as are illustrated in Fig. 4.

### Alteration in reward system

Our research showed that MOH patients have increased gray matter in the bilateral ventral striatum and ventral tegmental area (VTA) gray matter, which indicates that addiction caused by an abnormal reward system constitutes important pathogenesis of MOH. The ventral striatum contains nucleus accumbens, a pivotal dopaminergic area in the brain system related to reward processing. It receives signals from the orbitofrontal gyrus, the anterior cingulate gyrus, and the midbrain (Haber & Knutson, 2010). Research on non-human primates has identified nucleus accumbens, dorsal striatum, and orbitofrontal cortex are key areas responsible for the regulation of reward. Comfortable stimuli such as food or expectation of benefit activates these brain areas, which then release dopamine. Therefore, the nucleus accumbens is also referred to as the "pleasure center" (Di Chiara, 2002). Functional magnetic resonance imaging studies of adults also observed that the BOLD signal in these areas changes under reward conditions (May et al., 2004). The dysfunction of the reward system is considered to play a crucial role in drug abuse and addiction, for it’s been reported that the ventral striatum of patients with cocaine abuse has increased gray matter (Connolly et al., 2013). It is generally believed that drugs, which a patient relies on for reasons, can cause dopamine release from VTA by stimulating the γ-aminobutyric acid A receptors. Then dopaminergic receptors of nucleus accumbens received the signal from VTA and activates the reward system, prompting the patient to take drugs repeatedly. Structural and functional connection between VTA and nucleus accumbens was previously confirmed (Wang et al., 2019). Therefore, the alteration in the ventral striatum and VTA indicates a close connection between the reward system and MOH. Enlarged ventral striatum could work as a biomarker for MOH. Despite this potential association, more neuroimaging evidence is required, for enlarged nucleus accumbens was also found in CM patients (Planchuelo-Gómez et al., 2020).

Combined with the results of other clinical studies and functional imaging studies, addiction is considered to be proven to be involved in the pathogenesis of MOH. The drug overuse behavior of MOH patients is similar to that of other substance dependence such as drugs. As was estimated that about 2/3 of MOH patients met the diagnostic criteria for drug dependence (DSM-IV) (Radat et al., 2008). Functional MRI discovered brain dopamine system dysfunction in MOH patients relative to HCs, and it returned to normal after six months of drug withdrawal (Ferraro et al., 2012).

The important pathogenesis of substance addiction of MOH inspires us to treat MOH through behavioral intervention. This also corroborates that withdrawal of analgesics and replacement with non-addictive prophylactic medication is effective in relieving pain for most MOH, and preventing medication overuse in patients with other chronic headache who are vulnerable to drug abuse.

### Alteration in orbital frontal gyrus

Our results suggested the presence of gray matter atrophy in prefrontal cortex in MOH patients, including orbitofrontal cortex (OFC), superior frontal gyrus, middle frontal gyrus, and inferior frontal gyrus. Gray matter atrophy of the orbital gyrus passed FWE correction. Given that gray matter atrophy may indicate neural degeneration or dysfunction, this finding echoes with a previous PET study (Fumal et al., 2006) which found hypometabolism in the orbitofrontal gyrus on MOH patients relative to HCs.

Firstly, apart from gray matter decrease, there was also convincing evidence based on FDG-PET behavioral and pharmacological studies (London, 2000; Volkow et al., 2004) that OFC plays a vital role in drug addiction, craving, and compulsive behavior, which are associated with abnormally activated striatum-thalamus-orbitofrontal cortex circuit. The abnormality of OFC affects people’s expectations and desires, (Schoenbaum & Roesch, 2005) while weakens decision-making capabilities, for which MOH patients are more susceptible to essentially ineffective analgesics.

Secondly, OFC is believed to be associated with depression. The VBM study (Webb et al., 2014) found that the severity of depressive symptoms was related to the reduction of gray matter in areas such as OFC. Given that depression and anxiety often co-exist with MOH, we speculate that depression may be a risk factor for overuse of drugs. This may explain why amitriptyline, as an antidepressant, is recommended as a prophylactic medication for migraine. It was found that, for migraineurs who overdose analgesics, amitriptyline can reduce their dosage of analgesics and frequency of headache attacks (Hering & Steiner, 1991).

Abnormality OFC is widely considered characteristic of MOH (Riederer et al., 2012). Increase of gray matter volume of OFC might predict better treatment response (Lai et al., 2016). Nonetheless, further evidence is required to support such an idea. Two MRI studies (Beckmann et al., 2018; Chanraud et al., 2014) (17MOH vs 17 normal, 27MOH vs 27 normal, respectively) did not find significant gray matter differences in MOH comparing to HCs. Another PET study (Di et al., 2013) (10MOH vs 17 normal) reported no significant metabolic change in the orbitofrontal gyrus in MOH patients. Facing these divergent findings, we need to, on the one hand, pay attention to their relatively small sample sizes that were more susceptible to random errors. In this regard, future neuroimaging studies may require a larger sample size to confirm whether OFC anomalies are consistent in MOH. On the other hand, the prefrontal cortex itself, especially the orbitofrontal gyrus, is involved in a variety of neurological functions. With the help of data-driven Meta-Analytic Connectivity Modeling and using OFC as a seed region of interest, OFC (Zald et al., 2014) was found with extensive functional connections with multiple brain areas such as the default mode network, and participates in functions such as language, memory, and emotion. Therefore, it can be speculated that OFC alteration may present a relationship with other comorbid neuropsychological disorders. One piece of advice for future MOH-related neuroimaging studies is that subjects’ comorbid neuropsychological diseases be more rigorously investigated and controlled as confounding factors, so that more affirmative findings can be obtained.

### Alteration in pain network

Our results suggest that changes in multiple regions associate with the perception of pain. Periaqueductal gray (PAG) and trigeminal afferent area in the midbrain, thalamus and cerebellum, reported gray matter increase in MOH, whereas insula reported gray matter decrease. Notably, the second synthesis shows gray matter in midbrain and cerebellum shrank back after medication was ceased (see Table 5).

Pain processing is recognized as a highly complex process that involves multiple brain areas. After a person is stimulated by pain, cerebral hemodynamic changes and functional MRI activation was observed in the primary and secondary somatosensory cortices, the anterior cingulate gyrus, and the insula, which were thereafter named the pain matrix (Peyron et al., 2000; Porro, 2003). Midbrain PAG, another crucial region of the pain processing network, has been shown to be closely related to the downward inhibition of pain sensing (Millan, 2002). The insular cortex, in which lesion can cause hyperalgesia (Starr et al., 2009), is an anatomical interface between afferent processing and cognitive modulation systems. Our study suggests that MOH is closely related to a reversible modification in pain network. There were also functional evidence supporting such finding (Grazzi et al., 2010). Nonetheless, gray matter hyperplasia in the midbrain may not be unique to MOH, since it is also present in migraine (May, 2009). To better demonstrate the effect of medication overuse, further comparative studies between MOH and other primary headache disorders are in need, such as episodic migraine, which is less associated with drug abuse.

Moreover, our research confirms that MOH is also closely linked to cerebellar gray matter increase, which normalized after analgesics withdrawal. Meanwhile, PET study observed increased glucose metabolism in the cerebellar vermis in MOH that also normalized after analgesic withdrawal (Fumal et al., 2006). Cerebellum is found to be involved in pain perception and cognition by neuro-anatomic studies. In animal experiment, it was found that pure C-fiber reached the cerebellum (Jie & Pei-Xi, 1992). Such association between MOH and cerebellum was also supported by fMRI evidence. Compared with normal controls, there was a significantly enhanced functional connectivity between the left cerebellar hemisphere and the midbrain peri-aqueduct gray matter in MOH patient (Michels et al., 2017). Clinical study showed that patients in the recovery period of cerebellar infarction are more sensitive and severer to mechanical and thermal stimulation than normal people (Ruscheweyh et al., 2014).

Besides, due to insufficient functional neuroimaging studies on MOH, ALE analysis with either fMRI or PET was not feasible, despite their significant neurological value. Nevertheless, available studies found that MOH reported functional change in the pain network comparing to HCs. Multiple functional connectivity abnormalities was found in the trigeminal spinal tract nucleus, operculum, secondary somatosensory cortex (Mehnert et al., 2018). Hypermetabolism in insula was reported (Di et al., 2013).

## Conclusions

This meta-analytic ALE confirmed that MOH is associated with morphologic alteration in the reward system, the prefrontal cortex and a reversible modification in the pain network. Further functional imaging paradigms and longitudinal studies are required for a more definite conclusion and a causal mechanism.

### Limitations and prospects

Our study is limited by insufficient data available. Original neuroimaging studies on MOH included are few. One reason for this is that divergence in imaging data analysis techniques shrank the number of studies that can be combined. As an example, rest-state BOLD-based fMRI alone can be analyzed by rest-state-based amplitude of low frequency fluctuation(ALFF), ReHo, whole brain independent component analysis(ICA), and region of interest(ROI)-based functional connection, functional connection density(FCD), not to mention tasks-based fMRI studies. Limited number of comparable studies weakens the power of conclusion and brings out another limitation that most results are presented with uncorrected *P* < 0.001. Although uncorrected coordinates are supported by only one study, the pooled results may reflect a more generalized pattern after combining findings from different studies.

From the prospective the control selection, we identified only two studies comparing detoxified headache patients with MOH. More similar studies may help distinguishing reversibility in cortical alterations. Furthermore, few studies compared CM versus MOH. CM patients are susceptible to MO, thus constituting the major type of primary headache disorder of MOH. Some of the altered brain regions, e.g. enlarged nucleus accumbens (May, 2009; Planchuelo-Gómez et al., 2020) and structural alteration in orbitofrontal gyrus (Mehnert et al., 2018; Planchuelo Gómez et al., 2021), are shared by both CM and MOH. To observe the sole neural impact associated with medication overuse, more studies comparing CM with MOH is needed.

Notably, overused medication could provide crucial information. The comparisons between pre- versus post-withdrawal of medications, and MOH versus chronic headaches (e.g. migraine), may also be invaluable, for they may demonstrate the effect of medication overuse specifically. Although such pooling was infeasible due to limited articles, it is critical in explaining the unique pathophysiology of MOH. In addition, overused medications were diverse and overlapping in the included studies, so we were not able to perform subgroup analysis on types of drugs. To explore respective patterns of grey matter alterations according to overused drugs may be useful in elucidating MOH pathogenesis because pharmacology of different medication, e.g. migraine-specific ergots and triptans, opioids, and anti-inflammatory drugs, may indicate pathogenesis of MOH. For example, anti-CGRP monoclonal antibody, which cannot pass blood brain barrier, proved effective on MOH (Sun-Edelstein et al., 2021), implying that peripheral trigeminal nociceptor activation is a crucial mechanism in maintaining the headache in MOH.

We also found that the sample size of most neuroimaging studies was insufficient. It’s worth noting that all five included studies had sample sizes of less than 40 per group, which could bring in considerable random error and undermined the stability of the results, impairing the quality and reliability of conclusion.

Furthermore, given that MOH is a chronic disease, neuroimaging paradigms may also focus on ictal periods, so as to capture neuropathological activities during headache attacks. Notably, studies published so far are mostly in case–control design, which cannot prove causality. We have not identified any temporal study observing how chronic headaches develops into MOH, and how these neuroimaging changes occur. Longitudinal or cohort studies may hopefully disperse the mist clouding the pathogenesis of MOH.

In addition, acupuncture, a non-pharmaceutical treatment for chronic headaches, is worth research attention. It was reported that acupuncture could decrease the mean monthly number of moderate/severe migraine days compared with topiramate (Yang et al., 2011). Being a prophylactic measure, acupuncture may benefit MOH patients in both medication cessation and pain relief. There have been a variety of neuroimaging studies on the effects of acupuncture on primary headaches, but not on MOH yet.

In terms of morphometrics, cortical thickness (CT) and local gyrification index (LGI) calculation are also informative gray matter morphometrics. They may supplement VBM from a surface-based prospective. One study, which compared CT and LGI of MOH brains with healthy ones, found reduced CT in the left prefrontal cortex. Also, higher LGI was observed in fusiform gyrus and right occipital pole, the latter of which predicted poor response after detoxification.

Regarding the statistical methodology of ALE, given the coordinate-based data requires only the peaks of altered brain regions. The volume, shapes and sizes of the regions depicted in the original study are omitted, even though they contain valuable information. Furthermore, unlike the traditional META analysis, the ALE method is unable to incorporate negative findings (i.e. studies reporting no group-wise difference, yielding zero vertex) into the statistical analysis. Such negative reports, if included in the algorithm, may improve the comprehensiveness of our ALE results.

Last but not least, the interpretation of neuroimaging findings requires more evidence. The histological implication of gray matter alteration is so far uncertain and even controversial. It was found that the reduction of brain gray matter in patients with fibromyalgia is not accompanied by damage to the structural or functional integrity of neurons (Pomares et al., 2017). The increase in gray matter can be explained by the up-regulation of GABA receptors and possible inflammatory edema, rather than neuron increase. Interpreting brain gray matter reduction as neuronal damage or synaptic loss lacked cytohistologic evidence (May, 2009). Therefore, cytohistologic methods, electro-encephalogram, other modalities of MRI including magnetic resonance spectroscopy and diffusion tensor imaging, are much needed for a deeper understanding into MOH.

## Data Availability

All data and materials that support this manuscript are available at request.

## Code availability

Software used in this research is publicly available.
